# Assessment of knowledge, attitudes, and practice regarding antibiotic self-treatment use among COVID-19 patients

**DOI:** 10.3205/dgkh000415

**Published:** 2022-07-01

**Authors:** Hassan Mahmoudi

**Affiliations:** 1 Department of Microbiology, Hamadan University of Medical Sciences, Hamadan, Iran; 2 Assistant Professor of Medical Bacteriology, Nahavand School of Allied Medical Sciences, Hamadan, Iran

**Keywords:** knowledge, attitude, practice, self-medication, antibiotics, COVID-19

## Abstract

**Background:**

Self-medication with antibiotics is a common practice across different age groups and different cultures that can cause problems such as drug resistance, side effects, and rising costs for the healthcare system community. During the coronavirus pandemic (COVID-19), people with mild symptoms have avoided seeing a doctor, preferring to self-medicate. The impact of self-medication in COVID-19 patients is an important public health issue. The aim of this study was to evaluate the knowledge of, attitudes toward, and performance of self-medication with antibiotics in COVID-19 patients.

**Methods:**

This cross-sectional study was conducted among patients with COVID-19. Participants were selected by random sampling. A survey of knowledge, attitude and practice of taking antibiotics in patients with COVID-19 was conducted. Data were analyzed using SPSS version 22 software.

**Results:**

The prevalence of self-medication with antibiotics in patients with COVID-19 was 56.1%. Most COVID-19 patients have the overall knowledge, attitude and practice score of self-medication with antibiotics. There was a significant difference between the knowledge and attitudes of educated and uneducated patients (p<0.01). Of the demographic variables, there was no significant difference between sexes in terms of attitude and practice of antibiotic self-medication in COVID-19 patients (p>0.05).

**Conclusion:**

Considering the high prevalence of antibiotic self-medication in COVID-19 patients, it is recommended to provide the necessary education and practical means of reducing the amount of antibiotic self-medication.

## Zusammenfassung

**Zielsetzung:**

Die Selbstmedikation mit Antibiotika ist in verschiedenen Altersgruppen und Kulturen gängige Praxis, die Probleme wie Arzneimittelresistenzen, Nebenwirkungen und steigende Kosten für das Gesundheitssystems mit sich bringen kann. Nach dem Auftreten von COVID-19 haben Menschen mit leichten Symptomen den Gang zum Arzt vermieden und vorgezogen, sich selbst zu behandeln. Die Auswirkungen der Selbstmedikation bei an COVID-19 Erkrankten ist ein wichtiges Thema für die öffentliche Gesundheit. Ziel der Studie war es, Wissen, Einstellung und Verhalten von an COVID-19 Erkrankten in Bezug auf die Selbstmedikation mit Antibiotika zu bewerten.

**Methode:**

Die Querschnittsstudie wurde bei an COVID-19 Erkrankten durchgeführt. Die Teilnehmer wurden nach dem Zufallsprinzip ausgewählt. Es wurde eine Umfrage zu Wissen, Einstellung und Praxis der Antibiotikaeinnahme durchgeführt. Die Daten wurden mit der Software SPSS Version 22 ausgewertet.

**Ergebnisse:**

Die Prävalenz der Selbstmedikation mit Antibiotika bei Erkrankten mit COVID-19 betrug 56,1%. Die meisten Patienten waren in Bezug auf die Selbstmedikation mit Antibiotika durchschnittlich informiert. Es bestand ein signifikanter Unterschied zwischen dem Wissen und der Einstellung gebildeter und ungebildeter Patienten (p<0,01). Zwischen demographischen Variablen und Geschlecht gab es keinen Unterschied (p >0,05).

**Schlussfolgerung:**

In Anbetracht der hohen Prävalenz der Antibiotika-Selbstmedikation bei an COVID-19 Erkrankten wird eine Aufklärung einschließlich praktischer Angebote empfohlen, um die Selbstmedikation mit Antibiotika zu reduzieren.

## Background

Self-medication is a common practice across all ages and cultures, it being the arbitrary selection and use of a drug that one finds perfectly appropriate for one’s health. However, this is a global issue of concern [1], [2]. In general, people tend to be self-affirming, despite adequate knowledge about the importance of appropriate use of antibiotics [3]. This leads to inadequate treatment outcomes and the concomitant waste of resources, antibiotic resistance, and ultimately serious health risks [4]. During the COVID-19 pandemic, people with mild symptoms have avoided seeing a doctor, preferring to self-medicate [5]. Furthermore, the COVID-19 pandemic situation presents potential threats that could impact antimicrobial stewardship activities and lead to antibiotic resistance. For example, most people with COVID-19 have mild illness without pneumonia or moderate illness with pneumonia are given antibiotics. One of the factors that influence substance abuse is the purchase of over-the-counter antibiotics to take at home. Sharing medications with other people and prescribing more medications than patients need are additional factors that play a significant role in antibiotic abuse [6], [7]. Iran is one of the largest consumers of antibiotics in the world due to rampant self-medication. In addition, studies have shown that the amount of medication prescribed, particularly by general practitioners, is excessive, leading to storage of medication at home and eventual overuse. Substance abuse per se is also a major problem worldwide [8], [9]. One of the serious complications of improper use of antibiotics is antibiotic resistance, which is one of the common and unique challenges that has led to the emergence of resistance in pathogenic bacteria and their evolution. New antibiotics are not able to compete with antibiotic-resistant bacteria. As a result, it can be said that the increase in mortality in society is one of the consequences of antibiotic resistance [9]. The increase in mortality in the general population is a consequence of antibiotic resistance. The purpose of this study was to increase understanding of the relationship between the different causes of the spread of resistance and evaluate the antibiotic treatment process to extract important therapeutic strategies which reduce antibiotics risks [10].

## Material and methods

This cross-sectional study analyzed the knowledge of attitude towards and practice of self-medicating with antibiotics in COVID-19 patients. 450 patients with confirmed SARS-CoV-2 infection were evaluated according to the inclusion criteria of the study. These patients were admitted to Ayatollah Alimoradian Hospital affiliated with Hamadan University of Medical Sciences (UMSHA), Nahavand Province, Iran. The study was conducted from 11 March 2020 to 13 October 2020.

In addition, a demographic survey tool was used to measure knowledge, attitudes, and practice of COVID-19 patients based on the 30-item questionnaire, which consisted of three sections. The first part examined the knowledge of the COVID-19 patient on antibiotics, antibiotic resistance and antibiotic use. This section contained ten questions with possible answers being “true”, “false”, and “don't know”. A score of one was given to the correct answer and zero to the incorrect answer or “do not know” the answer. The maximum qualifying score is 10 and the minimum section score is zero. In general, scores above 5 were recognized as high knowledge level and below 5 as low knowledge level.

The second part, which includes ten questions, focuses on patient’s attitudes toward the choice and patterns of antibiotic use. Two questions from the set of attitude questions are expressed as a percentage and the rest are as a five-point Likert scale. The maximum and minimum scores obtained in this section are 40 and 8, respectively.

The third section, which consisted of ten questions, was to assess patient’s performance on antibiotics, which assessed patient’s behavior regarding the use of antibiotics and compliance with the dose and duration of treatment. The maximum and minimum scores obtained in this section are 45 and 9, respectively.

Overall, above-average scores were considered a high performance, and below-average scores were considered low performance. In addition, the total score was calculated from the answers to the three parameters knowledge, attitude and practice, with a maximum score of 95 and a minimum score of 17. For quantitative analysis, scores above 80% were good, from 60% to 80% intermediate and below 60% were considered poor. This questionnaire is valid and reliable for recording knowledge, attitudes, and performance in the field of antibiotic use [2], [11]. In this study, Cronbach’s alpha was used to assess reliability. Cronbach’s alpha for knowledge, attitude, and practice were 0.793, 0.812, and 0.832, respectively. Data were analyzed using SPSS version 22, and due to the non-normality of the data, the nonparametric Chi-Square, Kruskal-Wallis, and Mann-Whitney tests were performed. p<0.05 was considered statistically significant. 

## Results

Out of 450 people, 436 had completed the questionnaires. 14 questionnaires were incomplete and excluded from the study. The demographic variables and the difference between the mean scores of knowledge, attitude and practice of patients performing self-medication with antibiotics are shown in Table 1 (Tab. 1). 

### Knowledge

The average knowledge score was 6.0. 20% of patients had poor knowledge about antibiotics. 61.5% of COVID-19 patients responded that antibiotics are not appropriate for viral infections. 62.5% of COVID-19 patients believed that antibiotics for sore throat and dry cough can help prevent COVID-19.

### Attitude

The mean of the patients' attitudes was 28, the maximum value was 40. 24.7% of the COVID-19 patients had a positive attitude towards antibiotics. There was no significant difference by gender for knowledge, attitude, or practice, but there were significant differences according to educational level. The results showed that 31.2% of women and 38.9% of men had a positive attitude about antibiotics. The results showed that 25.9% of patients with COVID-19 have a positive attitude towards antibiotic treatment.

## Discussion

The study was conducted on 436 patients with COVID-19 to assess knowledge, attitude and practice of self-medication with antibiotics. The results showed that the prevalence of antibiotic treatment was 56.1%. Most patients who self-medicate with antibiotics have low awareness, low attitude, and average practice. Mamo et al. [12] showed that almost half (50.6%) of the study participants have a good knowledge of general drug use regarding COVID-19 and 65.4% had a positive attitude to general drug use regarding the treatment of COVID-19. Sarraf et al. [13] identified approximately (57.9%) of study participants with high knowledge.

56.1% of respondents in our study answered that the use of antibiotics to treat COVID-19 is beneficial. There was no association between gender and antibiotic use. 19.5% of the patients stated that a viral infection is cannot be treated with antibiotics. Heydargoy [5] reported on the use of antibiotics during the COVID-19 pandemic, finding that 38.1% of the participants used antibiotics without a doctor's prescription before the outbreak of COVID-19. In comparison, 20.8% of the participants used antibiotics during the COVID-19 epidemic, 20% said the COVID-19 outbreak and quarantine prevented them from seeking medical treatment, which is why they preferred self-medication. Thus, it can be concluded that the COVID-19 pandemic in Iran has increased the use of antibiotics. Due to the fear of leaving home and going to crowded places, especially medical centers that could be affected by COVID-19, people are staying at home and tend to self-medicate [5]. 

In terms of attitudes toward antibiotic use, Nepal et al. [14] showed that almost half of the respondents (47.7%) still believed that “antibiotics help them recover faster when they have a fever”. 

Although respondents knew that antibiotic resistance is a problem, half were still unsure whether taking antibiotics to prevent COVID-19 could increase antibiotic resistance. 

The only sociodemographic factor associated with knowledge, attitudes and practices related to antibiotic use was education. Respondents with higher education had better knowledge and more appropriate attitudes and practices, a finding which is consistent with other studies [15], [16], [11]. Our results also suggest that respondents in urban areas had better knowledge about antibiotic use than those in rural areas, a similar to observations made in the Nepal study [14].

Our study provides an evidence base from which to develop educational programs for the community about antibiotic use. For example, since several respondents could not identify antibiotics as posing a similar threat as other drugs, educating the public about the functions of antibiotics and the ability to distinguish them from other drugs could help minimise the misuse of antibiotics. 

The concept of antibiotic resistance is well known, but the issues associated with antibiotic misuse are not well understood. This study also showed that the community has high expectations of prescription antibiotics, increasing the likelihood of over-the-counter antibiotic use. Village doctors or health workers could educate community members and conduct mass awareness campaigns to highlight the potential risks of resistance to using antibiotics without a prescription and the inappropriateness of using antibiotic therapy for minor ailments. The study also identified an association between respondents with less knowledge, less appropriate attitudes and poor practices regarding the proper use of antibiotics.

## Conclusions

It is suggested that medical organizations and authorities reduce the phenomenon of self-medication and face-to-face visits during virus outbreaks by expanding virtual consultation systems with doctors and reducing the cost of these services, as well as introducing and teaching how to use this tool. The need for educational intervention and public legal limits for the use of antibiotics sold without a prescription is urgent.

## Notes

### Declarations

The project was approved by the Ethics Committee of Hamadan University of Medical Sciences with the ethics code (IR.UMSHA.REC.1399.914). The study on humans was conducted in accordance with the ethical standards of the Helsinki Declaration and Good Clinical Practice. 

### Funding

This research (ID: 991148144) was supported by Vice Chancellor for Research & Technology of Hamadan University of Medical Sciences, Hamadan, IRAN. 

### Conflict of interest

The author declares no conflict of interest, financial or otherwise. 

## Figures and Tables

**Table 1:**
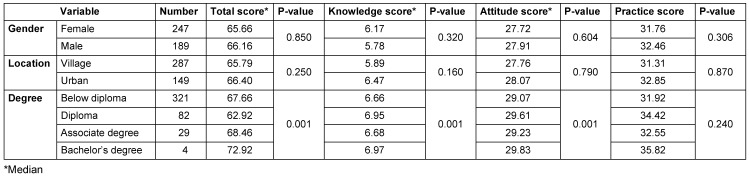
Mean score of knowledge, attitude and practice of patients self-medicating with antibiotics based on demographic variables
